# Progress in Cancer

**Published:** 1903-12-19

**Authors:** 


### Progress in Cancer.

Robert Abbe[1] considers the differentiation of breast tumours as far as the cysts are concerned, and points out from his own private experience of 41 cases of cyst and 56 cases of scirrhus that position is an important element in diagnosis. (1) Cysts are localised in any part of the gland, with slight predominance only in upper and outer segment. (2) Scirrhous tumours are almost universally distributed between the nipple and axilla. The position of the latter may show some progressive advancing absorption of some infective cause, having entrance at the nipple and advancing along the line of main lymphatic currents. He states from these considerations that a tumour in the lower half of the breast has the greater probability of being a cyst. The aspirating needle should always be used in any case of doubt, and as aspiration is, in his experience, sufficient to cure cysts, he urges that a barrel should be used in the syringe capable of holding two drachms or so, and thus exhaust the cyst with one puncture.

Robert Bell, after briefly recording the views advanced as to the causation of cancer,[2] acknowledges that he does not feel convinced that any of these is reconcilable with the truth. He considers that were this disease due to a parasite or microbe, the incidence would be marked by a rise of temperature such as would be naturally produced as the result of the irritation excited. Again, it would not be found to be unilateral, as it is almost invariably at the onset. He believes the cause to be a "normal cell which from a combination of circumstances, has become altered in character, and has assumed a new *rôle* of existence." This cell proceeds to carry on a separate existence, preying upon its neighbours and living and dividing at their expense. Every circumstance which tends to impoverish or militate against the general health predisposes to cancer, in that it lowers the resistance of the healthy cells to the ravages of the morbid cell. An injury to an organ which has already been the seat of a esion, or has suspended its physiological activity is especially a predisposing cause. Cancer is practically unknown among savages and those leading a "natural" life. The integrity of the epithelium depends largely upon the unimpaired integrity of the thyroid body. This body, according to Blumm, seizes upon and renders innocuous certain toxic substances absorbed from the intestine into the blood. Its use in cancer cases depends upon its action on the toxins due to the condition of constipation frequently present, and the over-indulgence in nitrogenous diet; also in the second place the coincident presence of the saccharomycetes in the intestines causes fermentative changes in these toxins and a condition of uric-acidæmia produced. This again is a serious predisposing cause of cancer, interfering as it does gravely with cell metabolism. These entero-toxins will account for the cachexia. He urges that since certain animals fed on a milk diet after thyroidectomy will remain in good health, and will die if meat is substituted, so milk should be mainly substituted for the more richly nitrogenous foods in the cancer patient. The toxins formed from a milk diet differing probably in quantity rather than quality from those produced by a meat diet. The thyroid gland, with its power of neutralising these toxins, should be administered as an antidote, and the salicylates given as well to combat the saccharomycetes or the cause of the uric acid production. The bowels are also to be daily evacuated. He recommends thyroid extract gr. v. thrice daily, sodium salicylate gr. x.–xv. thrice daily and the treatment of local affections to remove any irritation present. He is hopeful that cases that have not advanced too far may yet be relieved by this method of treatment.

From a study of the population of India a writer in the *London Polyclinic*, May,[3] discusses the causation of cancer. Amongst the Mussulman population, as with the Jews, where circumcision is practised, cancer of the penis is rare ; while with the Hindoos, who have long foreskins, cancer is common of the penis. The mouth is frequently affected through the irritation of the betel-nut chewing, though not the lips, smoking not being a frequent custom and the short clay pipe not being used. Cancer is also found on the abdomen due to the habit of carrying a portable earthen brazier under the clothes. These three positions form the commonest situations for cancer incidence in India ; and it is difficult, says the writer, to see how these three varying causes can be due to a parasitic contagion.

D'Arcy Power [4] has observed that some patients after operation for malignant disease, if relieved of anxiety and if they travel or reside in another part, may lose their disease or show recurrence much later than would be supposed from the nature of their disease. Also, that cases as nearly similar to the foregoing as can be, returning to their old home, or undergoing worry, show recurrence early and die soon. He invites the collection of statistics as to whether this is in the nature of a frequent phenomenon, and as to whether it is justifiable to recommend such patients to change their mode and place of living as completely as may be.

[1] Med. Rec., Aug. 15. [2] Ibid. [3] Ibid., July 4. [4] Lancet, July 25.

*(To be continued.)*

